# METTL3 Regulates Osteoblast Differentiation and Inflammatory Response via Smad Signaling and MAPK Signaling

**DOI:** 10.3390/ijms21010199

**Published:** 2019-12-27

**Authors:** Yiwen Zhang, Xiaofei Gu, Di Li, Luhui Cai, Qiong Xu

**Affiliations:** Guanghua School of Stomatology & Guangdong Provincial Key Laboratory of Stomatology, Sun Yat-sen University, Guangzhou 510055, China; zhywen@foxmail.com (Y.Z.); guxf3@mail2.sysu.edu.cn (X.G.); lidi5@mail2.sysu.edu.cn (D.L.); cailh6@mail2.sysu.edu.cn (L.C.)

**Keywords:** METTL3, osteoblast differentiation, inflammatory response, Smad, MAPK, mRNA stability, YTHDF2

## Abstract

Osteoblasts are crucial bone-building cells that maintain bone homeostasis, whereas inflammatory stimuli can inhibit osteogenesis and activate inflammatory response. N6-methyladenosine (m^6^A) is the most abundant mRNA modification in eukaryotes and plays important roles in multiple biological processes. However, whether m^6^A modification affects osteoblast differentiation and inflammatory response remains unknown. To address this issue, we investigated the expression of the N6-adenosine methyltransferase METTL3 and found that it was upregulated during osteoblast differentiation and downregulated after LPS stimulation. We then knocked down METTL3 and observed decreased levels of osteogenic markers, ALP activity, and mineralized nodules, as well as Smad1/5/9 phosphorylation, in LPS-induced inflammation. METTL3 knockdown promoted the mRNA expression and stability of negative regulators of Smad signaling, *Smad7* and *Smurf1*, the same regulatory pattern identified when the m^6^A-binding protein YTHDF2 was silenced. Moreover, METTL3 depletion enhanced proinflammatory cytokine expression and increased the phosphorylation of ERK, p38, JNK, and p65 in MAPK and NF-κB signaling pathways. The increase in cytokine expression was inhibited after MAPK signaling inhibitor treatment. All data suggest that METTL3 knockdown inhibits osteoblast differentiation and Smad-dependent signaling by stabilizing *Smad7* and *Smurf1* mRNA transcripts via YTHDF2 involvement and activates the inflammatory response by regulating MAPK signaling in LPS-induced inflammation.

## 1. Introduction

Bone is a highly adaptive tissue with remodeling processes coordinated by bone-forming osteoblasts and bone-resorbing osteoclasts. Osteoblasts have osteogenic differentiation potential and are responsible for mediating bone metabolism through synthesizing and depositing extracellular matrix [1,2,3]. BMP/Smad signaling is fundamentally important in bone homeostasis, and canonical Smad-dependent signaling exhibits pivotal regulatory functions in osteogenic gene expression by phosphorylating the Smad1/5/9 complex [4]. The exquisite balance of bone homeostasis can be disrupted by many infectious diseases, such as osteomyelitis, osteoarthritis, and periodontitis [5,6,7]. Lipopolysaccharide (LPS) from the cell membrane of gram-negative bacteria is recognized as the main pathogenic factor positively related to infectious bone destruction. It has been reported that LPS can suppress osteoblastic differentiation by affecting multiple pathways, such as BMP/Smad signaling, Wnt/β-catenin signaling, and Notch signaling [8,9,10]. Moreover, LPS stimulates osteoblasts to produce various cytokines by activating mitogen-activated protein kinase (MAPK) signaling and nuclear factor-κB (NF-κB) signaling, thus contributing to the global systemic and local inflammatory responses [11,12].

N6-methyladenosine (m^6^A), which is dynamic methylation at the N6 site of adenosine, has been reported as the most prevalent internal mRNA modification in eukaryotes since its first description in the 1970s [13]. Recent studies utilizing high-throughput sequencing techniques after m^6^A RNA immunoprecipitation revealed its conserved enrichment near stop codons, 3′UTRs, and long internal exons [14]. The unique m^6^A distribution suggests its involvement in mediating downstream molecule functions, facilitating different stages of mRNA metabolism [15,16]. The reversible deposition of m^6^A occurs via a methyltransferase complex consisting of a core methyltransferase-like 3 (METTL3)/methyltransferase-like 14 (METTL14) catalytic heterodimer [17,18] and is reversed by demethylases, fat mass and obesity-associated protein (FTO), and ALKB homolog 5 (ALKBH5) [19,20]. Except for ‘writers’ and ‘erasers’, the modification is recognized and bound by m^6^A ‘readers’ in mammalian cells, mainly YTHDF1-3 and YTHDC1, 2, which are from YTH-domain family proteins, thus contributing to various biological processes, such as viral infections, tumorigenesis, and adipogenic differentiation [21,22,23].

Accumulating evidence has pointed out the importance of m^6^A methylation in cellular function and fate control, while aberrant changes in methyltransferases and demethylases result in malfunction or diseases [24,25,26]. A recent in vivo study demonstrated that the reduced m^6^A modifications in bone marrow mesenchymal stem cells (BMSCs) disrupt the osteogenic and adipogenic responses induced by parathyroid hormone, resulting in severe bone loss and excessive adipose accumulation [27]. Our previous study also showed that METTL3 loss inhibited the osteogenic differentiation potential of BMSCs [28]. Nevertheless, little is known about the role of METTL3 in osteoblast function during the inflammatory process. It is also unclear whether METTL3 affects the LPS-induced inflammatory response and its regulatory pathway in osteoblasts. Therefore, our study aimed to investigate the effect and underlying molecular mechanism of METTL3 on osteoblast differentiation and inflammatory reactions.

## 2. Results

### 2.1. LPS Treatment Induces an Inflammatory Environment and Inhibits Osteoblast Differentiation

**Figure 1:**
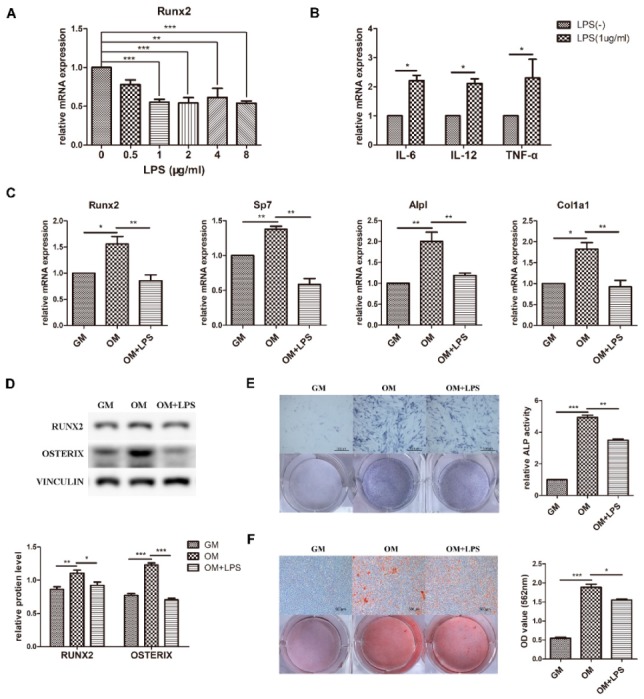
Effect of LPS on osteoblast differentiation and proinflammatory cytokine production. MC3T3-E1 cells were stimulated in osteogenic medium with or without LPS at different concentrations for 3–7 days. GM, growth medium; OM, osteogenic induction medium. (**A**–**C**) The mRNA expression of *Runx2, Sp7, Alpl, Col1a1, IL-6, IL-12*, and *TNF-α* was quantified on day 3 by qRT-PCR, and Gapdh was used as a normalization control. (**D**) The protein levels of RUNX2 and OSTERIX were measured on day 3 by western blotting. VINCULIN was used as an internal control. The band intensities were analyzed using ImageJ software. (**E**) ALP activity was assessed on day 7 using ALP staining. Scale bars, 500 μm. The growth medium group was used as an internal reference. (**F**) The formation of mineralized nodules was analyzed on day 7 using alizarin red staining. Scale bars, 500 μm. All the results are shown as the mean ± SD (*n* = 3). Significant differences were compared with the control or indicated group. The *p* values were calculated by one-way ANOVA. * *p* < 0.05, ** *p* < 0.01, *** *p* < 0.001.

Preosteoblast MC3T3-E1 cells were cultured in osteogenic medium to observe osteoblast differentiation ability. The results showed that the mRNA expression of the osteogenic markers *Runx2*, *Sp7*, *Alpl*, and *Col1a1* and the protein levels of RUNX2 and OSTERIX significantly increased (Figure 1C,D). Moreover, ALP activity and matrix calcium deposition were increased according to ALP staining and alizarin red staining, confirming the capability of osteoblast differentiation and mineralization (Figure 1E,F).

To investigate the effect of LPS-induced inflammation on osteoblast differentiation, expression of the key osteoblast transcription factor *Runx2* was detected after adding 0–8 μg/mL LPS to osteogenic medium (Figure 1A). The difference was statistically significant at a minimum concentration of 1 μg/mL, which was used for further experiments. The osteoblast inflammatory response was identified by the increased mRNA levels of *IL-6*, *IL-12*, and *TNF-α* (Figure 1B). As demonstrated in Figure 1C–F, the transcriptional expression and protein levels of the osteoblast markers decreased after LPS stimulation for 3 days, while ALP activity and mineralized nodules were decreased at day 7. These data showed that osteoblast differentiation was induced in osteogenic medium, simulating physiological osteogenesis, and inhibited in the LPS-mediated pathological inflammation environment.

### 2.2. m^6^A Methyltransferase and Demethylase Expression during Osteogenesis and Inflammation

**Figure 2:**
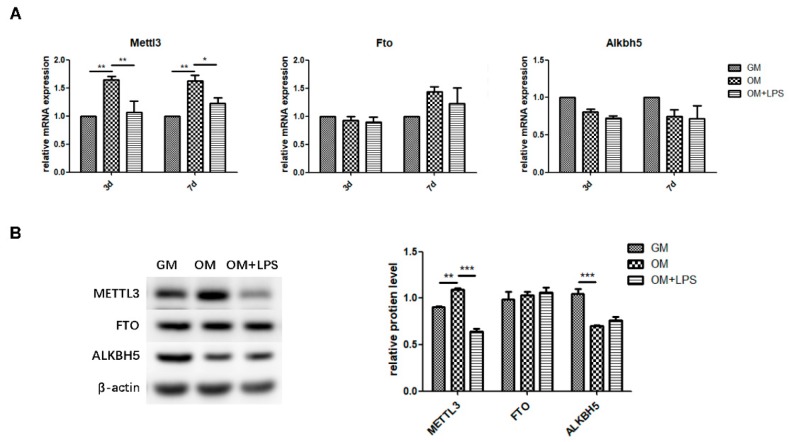
m^6^A methyltransferase and demethylase expression during osteogenesis and inflammation. MC3T3-E1 cells were cultured in osteogenic medium with 1 μg/mL LPS for 3 days. (**A**) The mRNA expression of *Mettl3*, *Fto*, and *Alkbh5* was quantified by qRT-PCR. Gapdh was used as an internal control. (**B**) The protein levels of METTL3, FTO, and ALKBH5 were assessed by western blotting and normalized to that of β-actin. The results are shown as the mean ± SD (*n* = 3). * *p* < 0.05, ** *p* < 0.01, *** *p* < 0.001.

To explore the role of m^6^A methyltransferase and demethylases in physiologic osteoblast differentiation and LPS-induced inflammation, we evaluated the expression patterns of METTL3, FTO, and ALKBH5. As measured by qRT-PCR and western blotting, METTL3 mRNA and protein levels increased during osteoblast differentiation and decreased after inflammatory stimulation, while the gene expression and protein levels of FTO were unchanged (Figure 2A,B). The mRNA expression of *Alkbh5* was not significantly different between the three groups (Figure 2A). Although ALKBH5 protein levels were reduced after osteogenic induction, they remained unchanged after LPS treatment (Figure 2B). Accordingly, the similar expression pattern of the m^6^A methyltransferase METTL3 and osteogenic markers implied that METTL3 might play a functional role in osteoblastic differentiation in the inflammatory environment.

### 2.3. METTL3 Knockdown Inhibits Osteoblast Differentiation and Mineralization in LPS-Stimulated Osteoblasts

**Figure 3:**
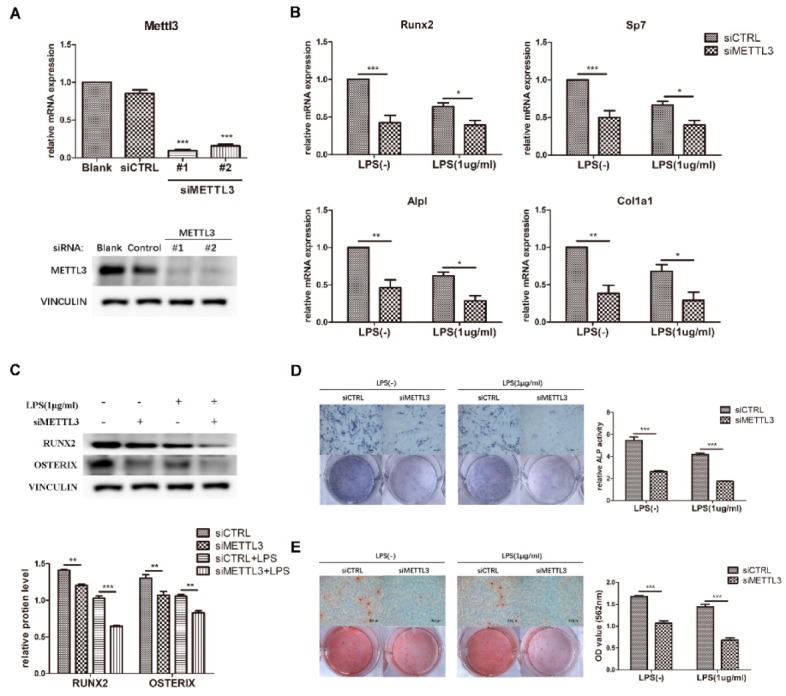
Effect of METTL3 knockdown on osteoblast differentiation. (**A**) The transfection efficiency of METTL3 knockdown was determined by qRT-PCR and western blotting. Blank, cells transfected with growth medium; siCTRL, cells transfected with negative control siRNA; siMETTL3, cells transfected with METTL3 siRNA. (**B**,**C**) MC3T3-E1 cells were transfected with siRNA (METTL3 or negative control) for 12 h and then treated with osteogenic induction medium with or without 1 μg/mL LPS for 60 h. The mRNA expression of *Runx2*, *Sp7*, *Alpl*, and *Col1a1* was measured by qRT-PCR (**B**). GAPDH was used as a normalization control. The protein levels of RUNX2 and OSTERIX were assessed by western blotting (**C**). VINCULIN was used as an internal control. The band intensities were quantified using ImageJ software. (**D**) The ALP activity of siCTRL or siMETTL3 cells undergoing osteogenic induction with or without LPS for 7 days was analyzed by ALP staining. Scale bars, 500 μm. The growth medium group was used as an internal reference. (**E**) The formation of mineralized nodules in siCTRL or siMETTL3 cells after osteogenic induction with or without LPS for 7 days was assessed by alizarin red staining. Scale bars, 500 μm. All data represent the mean ± SD (*n* = 3). * *p* < 0.05, ** *p* < 0.01, *** *p* < 0.001.

To determine the effect of METTL3 on osteogenesis and inflammation, cells were transfected with siMETTL3. Compared with those in the negative control group, METTL3 mRNA and protein levels were correspondingly decreased after gene knockdown (Figure 3A). siMETTL3 #1 yielded higher knockdown efficiency and was used in the following experiments.

To investigate the function of METTL3 in the osteoblast differentiation process, we detected the osteogenic potential after osteogenic induction for the indicated time in METTL3-knockdown cells. As demonstrated in Figure 3B, the mRNA expression of *Runx2*, *Sp7*, *Alpl*, and *Col1a1* apparently decreased in the siMETTL3-treated group, and the protein levels of RUNX2 and OSTERIX were decreased (Figure 3C). Consistent with the osteogenic marker expression results, ALP and alizarin red staining assays indicated that METTL3 knockdown inhibited ALP activity and mineralized nodule formation (Figure 3D,E).

LPS was then added to osteogenic medium, and the osteogenic markers were analyzed. Suppression of the osteoblast factors Runx2, Sp7, Alpl, and Col1a1 at the transcript and protein levels confirmed the effect of METTL3 knockdown on early osteoblast differentiation in the inflammatory environment (Figure 3B,C). Moreover, ALP activity and calcium deposition were inhibited in METTL3-knockdown cells after LPS treatment, verifying that METTL3 participated in osteoblast mineralization in LPS-stimulated inflammation (Figure 3D,E). Consequently, METTL3 acted as a positive regulator of osteoblast differentiation and mineralization in physiologic and inflammatory conditions.

### 2.4. METTL3 May Affect Smad-Dependent Signaling by Regulating Smad7 and Smurf1 mRNA Stability Via YTHDF2 Involvement

**Figure 4:**
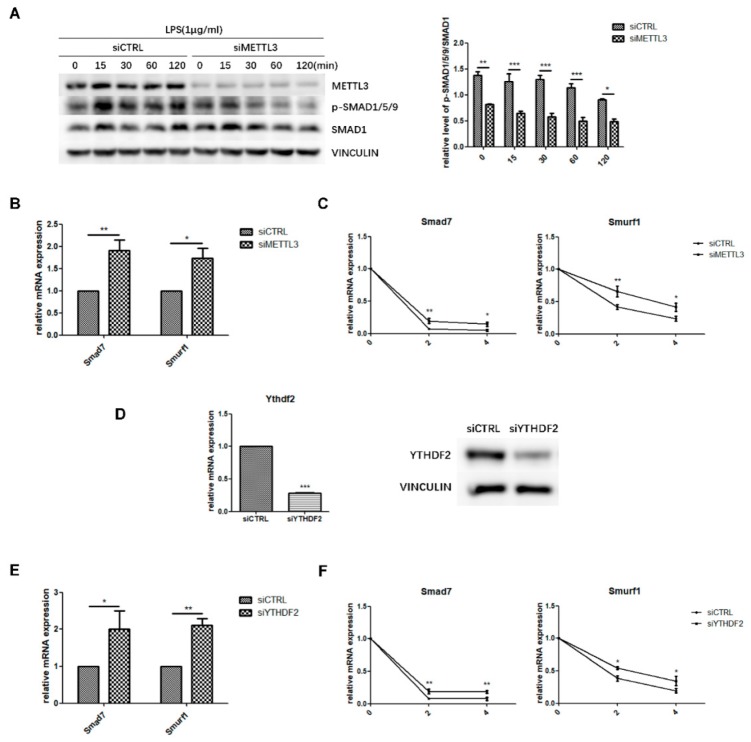
Effect of METTL3 or YTHDF2 knockdown on Smad-dependent signaling. (**A**) siMETTL3- or siCTRL-transfected cells were cultured in osteogenic medium for 60 h and then treated with 1 μg/mL LPS for 0–120 min. Next, Smad1/5/9 phosphorylation was examined by western blotting. p-Smad1/5/9/Smad1 values represent the relative level of signaling activation. VINCULIN was used as an internal reference. (**B**,**E**) MC3T3-E1 cells were transfected with siRNA (METTL3, YTHDF2, or negative control) and cultured in osteogenic medium for 60 h. *Smad7* and *Smurf1* mRNA levels were detected by qRT-PCR. Gapdh was used as an internal control. (**C**,**F**) The mRNA expression of *Smad7* and *Smurf1* was examined by qRT-PCR in siRNA (METTL3, YTHDF2, or negative control)-transfected cells with osteogenic induction and 0, 2, and 4 h of treatment with 5 μg/mL actinomycin D. Gapdh was used as an internal control. (**D**) The transfection efficiency of YTHDF2 knockdown was measured by qRT-PCR and western blotting. siCTRL, cells transfected with negative control siRNA; siYTHDF2, cells transfected with YTHDF2 siRNA. The results are shown as the mean ± SD (*n* = 3). * *p* < 0.05, ** *p* < 0.01, *** *p* < 0.001.

To explore the regulatory mechanism of METTL3 in osteoblast differentiation, we evaluated the canonical Smad-dependent signaling pathway, which is closely related to osteogenesis. As indicated by western blotting, the phosphorylation level of Smad1/5/9 significantly decreased in METTL3-knockdown cells after LPS stimulation (Figure 4A), indicating that METTL3 depletion has a negative effect on Smad signaling in LPS-stimulated osteoblasts.

Next, the expression of *Smad7* and *Smurf1*, two negative factors modulating Smad signaling, was analyzed to further study how METTL3 affected Smad signaling. We performed qRT-PCR and mRNA stability measurements using the transcription inhibitor actinomycin D to detect *Smad7* and *Smurf1* transcript half-lives. The results showed that METTL3 knockdown increased the expression level and mRNA stability of *Smad7* and *Smurf1* (Figure 4B,C). YTHDF2 is an m^6^A “reader” protein that preferentially recognizes m^6^A-containing mRNA and mediates its degradation [29,30]. Thus, we knocked down YTHDF2 and investigated whether it was involved in Smad signaling regulation (Figure 4D). As shown in Figure 4E, YTHDF2 depletion enhanced the mRNA levels of *Smad7* and *Smurf1*, while the transcript half-lives of *Smad7* and *Smurf1* had similar increases in the mRNA stability assay (Figure 4F). Overall, these data suggested that METTL3 depletion might inhibit Smad-dependent signaling activation by stabilizing *Smad7* and *Smurf1* mRNA via YTHDF2 involvement.

### 2.5. METTL3 Knockdown Activates MAPK Signaling to Promote Proinflammatory Cytokine Expression in LPS-Treated Osteoblasts

**Figure 5:**
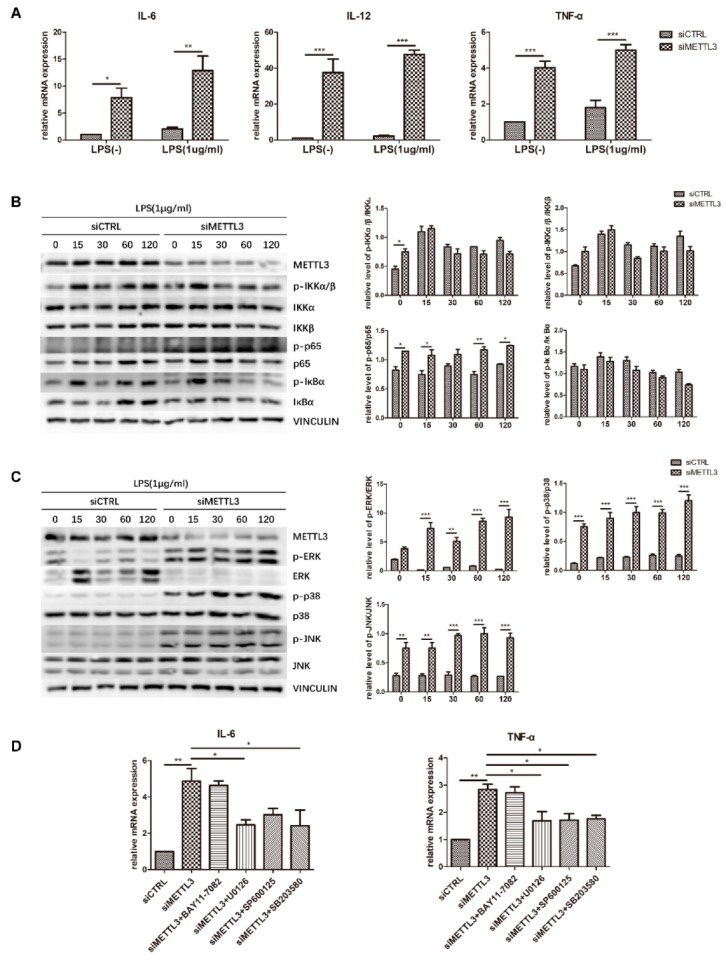
Effect of METTL3 knockdown on osteoblast proinflammatory cytokine production and MAPK and NF-κB signaling activation. (**A**) MC3T3-E1 cells were transfected with siRNA (METTL3 or negative control) and then cultured in osteogenic medium with or without 1 μg/mL LPS for 60 h. The expression of *IL-6*, *IL-12*, and *TNF-α* was examined by qRT-PCR. Gapdh was used as an internal control. (**B**,**C**) Cells transfected with siRNA (METTL3 or negative control) were cultured in osteogenic medium for 60 h and then treated with 1 μg/mL LPS for 0–120 min. MAPK and NF-κB phosphorylation was examined by western blotting, and VINCULIN was used as an internal reference. (**D**) Cells transfected with siRNA (METTL3 or negative control) were treated with the NF-κB inhibitor BAY 11-7082, the p38 inhibitor SB203580, the ERK inhibitor U0126, or the JNK inhibitor SP600125 for 1 h and then cultured in LPS-induced osteogenic medium for 60 h. The mRNA levels of *IL-6* and *TNF-α* were detected by qRT-PCR. Gapdh was used as an internal control. The results are shown as the mean ± SD (*n* = 3). * *p* < 0.05, ** *p* < 0.01, *** *p* < 0.001.

To identify the role of METTL3 in the osteoblast inflammatory response, cells transfected with siMETTL3 were induced with osteogenic medium with or without LPS, and cytokine gene expression was subsequently examined by qRT-PCR. The results showed that *IL-6*, *IL-12*, and *TNF-α* mRNA levels were remarkably increased in both METTL3-knockdown groups, even if the original expression was higher in LPS-treated groups than in non-LPS-treated groups (Figure 5A). This evidence confirmed that METTL3 inhibition boosts proinflammatory factor expression in osteoblasts.

To reveal the signaling pathway by which METTL3 regulates the inflammatory response, we evaluated NF-κB and MAPK signaling activation. Western blotting was performed to detect the phosphorylation levels of IKKα/β, p65, IκBα, ERK, p38, and JNK. The results showed that compared with the control condition, METTL3 depletion remarkably increased the levels of p-ERK, p-p38, p-JNK, and p-p65. In contrast, no obvious changes were observed in the other two components of NF-κB signaling, IKKα/β and IκBα (Figure 5B,C).

To further validate the effect of NF-κB and MAPK signaling activation on the inflammatory response regulated by METTL3, cells were pretreated with the NF-κB inhibitor BAY11-7082, the ERK inhibitor U0126, the JNK inhibitor SP600125, or the p38 inhibitor SB203580 to block these pathways. We assessed the mRNA levels of proinflammatory cytokines and found that the increases in IL-6 and TNF-α expression were limited after MAPK pathway inhibitor pretreatment but were not changed after NF-κB inhibitor pretreatment in METTL3-knockdown cells (Figure 5D). Consequently, we concluded that METTL3 depletion activates MAPK signaling to promote proinflammatory cytokine expression in LPS-treated osteoblasts.

## 3. Discussion

N6-methyladenosine, one of the most abundant mRNA chemical markers, is widely conserved in eukaryotic species ranging from yeast to humans [31]. The majority of m^6^A modifications are already evident in nascent pre-mRNA, where they are reversibly formed by the methyltransferase complex (METTL3, METTL14, and WTAP) and removed by demethylases (FTO and ALKBH5) [32]. Much evidence has showed that m^6^A modifications have an extensive influence on regulating alternative splicing, RNA stability, translation, and nuclear export with the assistance of m^6^A-binding proteins (YTHDF1-3, YTHDC1, 2) [16,33,34]. Currently, the vital function of m^6^A modifications in different physiologic and pathological processes, such as stem cell self-renewal and differentiation, immune responses, and tumorigenesis, has become a research hotspot in epitranscriptomics [24,35,36]. m^6^A methylation in mouse embryonic stem cells is reported to prevent HuR binding and destabilize the transcripts encoding developmental regulators to maintain a self-renewal state [37]. A METTL3 and METTL14 conditional knockout mouse study proved that m^6^A modification regulates T cell homeostasis by degrading SOCS gene family mRNA and alleviating IL-7 signaling blockade [38]. A recent finding revealed that m^6^A modification plays a negative role in the interferon response by regulating the fast turnover of interferon mRNA and promoting viral propagation [39].

Bone homeostasis is maintained by a balance between osteoblast-mediated bone formation and osteoclast-mediated bone resorption. Osteoblasts are the chief bone-building cells with the capability to regulate bone homeostasis [40]. Activated osteoblast differentiation is often characterized by the high expression of transcription factors, including Runx2 and Sp7, which subsequently affect the osteogenesis stage of production and mineralization of numerous extracellular matrix proteins, such as osteocalcin, ALP and type I collagen [1,3]. However, in disease conditions, differentiation is inhibited in infected osteoblast and osteogenesis is disordered, contributing to bone destruction [41,42,43]. Upon exposure to bacterial or viral infection, osteoblasts can directly respond to pathogen-associated molecular patterns (PAMPs), such as LPS, with the release of several cytokines, including IL-1β, IL-6, and TNF-α [12,44]. Although it has been reported that alterations in METTL3 modulate the osteogenic lineage commitment and differentiation of BMSCs and are involved in osteoporosis [27], the role of m^6^A modification in osteoblast differentiation in an inflammatory environment remains unclear. To elucidate whether m^6^A-dependent RNA methylation participates in osteoblast differentiation and inflammation, we cultured MC3T3-E1 cells in osteogenic induction medium with LPS to establish an inflammatory model. Osteoblast markers levels, ALP activity and mineralized nodules were increased in the osteogenic environment, validating the differentiation and mineralization ability of MC3T3-E1 preosteoblasts. In the LPS-induced inflammatory environment, the osteogenesis process was suppressed, and proinflammatory cytokine production was facilitated. The expression of the main m^6^A methyltransferase and demethylases, including METTL3, FTO and ALKBH5, was then measured. The results showed that only METTL3 expression increased after osteogenic induction and decreased upon inflammatory stimulation, indicating a close correlation between METTL3 and osteoblast differentiation and inflammation.

METTL3, a crucial m^6^A ‘writer’ protein with an S-adenosyl methionine-binding domain and methyltransferase capacity, was identified in the 1990s [45]. A recent study indicated that silencing METTL3 markedly promoted the porcine BMSC adipogenesis process by targeting the JAK1/STAT5/C/EBPβ pathway via an m^6^A-YTHDF2–dependent regulatory pattern [23]. METTL3 depletion suppressed the osteogenic differentiation potential of BMSCs and decreased the expression of *Vegfa* splice variants [28]. In the present study, to investigate the effect of METTL3 on bone-forming function in an inflammatory environment, the osteogenesis process was evaluated in siMETTL3 treated osteoblasts with or without LPS. The mRNA expression of osteogenic markers, including *Runx2*, *Sp7*, *Alpl*, and *Col1a1*, was obviously suppressed after METTL3 knockdown. Similarly, the protein levels of the transcription factors RUNX2 and OSTERIX, ALP activity, and mineralized nodule formation were also inhibited with or without LPS stimulation, which implied that METTL3 depletion down-regulated osteoblast differentiation and mineralization in both physiological and inflammatory environments.

It is generally acknowledged that the canonical Smad-dependent pathway is inextricably linked with the osteogenesis process. After binding to autocrine and paracrine bone morphogenic protein ligands, Smad-dependent signaling is activated during osteoblast differentiation. The Smad1/5/9 complex is phosphorylated and subsequently translocated with Smad4 into the nuclei, where they recruit RUNX2 to activate osteogenic gene expression [4,46]. Smad7 is an inhibitory Smad protein that negatively regulates Smad signaling by preventing Smad1/5/9 phosphorylation, degrading Smad1/5/9 via the ubiquitin proteasome with Smurf1, and inhibiting the translocation of complex nuclei [47]. To explore the molecular mechanism of the effect of METTL3 on osteoblast differentiation under inflammatory conditions, we examined Smad1/5/9 phosphorylation in METTL3-knockdown cells after LPS treatment. As shown by the data, the level of p-Smad1/5/9 decreased and the expression of two negative mediators, *Smad7* and *Smurf1*, increased in METTL3-inhibited cells, which is consistent with the reported Smad signaling regulatory pattern [48]. We hypothesized that METTL3 might participate in modulating osteoblast differentiation via Smad-dependent signaling in the inflammatory environment. Thus, we measured the mRNA stability of two negative factors and found that METTL3 depletion enhanced the half-lives of *Smad7* and *Smurf1*. YTHDF2 is a main m^6^A-binding protein that recognizes and destabilizes m^6^A-containing mRNA to control the expression of key genes in multiple biological processes [49,50]. Accordingly, we knocked down YTHDF2 and explored whether it is involved in modulating the negative mediators of Smad-dependent signaling. In our study, both the transcript expression and mRNA stability of *Smad7* and *Smurf1* increased in siYTHDF2 osteoblasts. Therefore, we speculated that METTL3 knockdown might suppress *Smad7* and *Smurf1* decay via YTHDF2 participation, resulting in Smad-dependent signaling blockage in inflammatory conditions.

Osteoblasts possess the capacity to respond to an infectious challenge by activating the MAPK and NF-κB signaling pathways to regulate the expression of downstream proinflammatory cytokines, such as IL-6, IL-12, and TNF-α. The secretion of these proinflammatory factors contributes to controlling the inflammatory response and creates an osteolytic environment, which might influence the activities of other bone cells [12,51]. To explore the function of METTL3 in the osteoblast inflammatory response, we examined the accumulation of proinflammatory cytokines and LPS-induced signaling pathways molecules in METTL3-knockdown cells. The expression of IL-6, IL-12, and TNF-α appeared to be significantly increased in both the physiological and LPS-stimulated microenvironments, indicating that METTL3 is involved in preventing the osteoblast inflammatory response. We also found that ERK, p38, JNK, and p65 phosphorylation in the MAPK and NF-κB signaling pathways was enhanced in METTL3-knockdown cells, further confirming the role of METTL3 as a negative regulator of the LPS-induced inflammatory response in osteoblasts. Signaling inhibitors were then used to further explore the effect of the NF-κB and MAPK pathways on the upregulated cytokine expression in METTL3-deficient osteoblasts. After blocking the signaling pathways, the increased levels of IL-6 and TNF-α were suppressed in the MAPK inhibitor groups but did not change in the NF-κB inhibitor groups. These results demonstrated that METTL3 regulates proinflammatory cytokine expression via the MAPK signaling pathway in LPS-treated osteoblasts. Given the correlation of bone homeostasis that maintained by a balance between osteoblasts and osteoclasts, METTL3-dependent m6A methylation might also play a role in osteoclast differentiation and activity. More studies are necessary to further elucidate the function of N6-methyladenosine in regulating bone homeostasis.

## 4. Materials and Methods

### 4.1. Cell Culture and Treatment

Preosteoblast MC3T3-E1 cells obtained from the National Infrastructure of Cell Line Resource (Beijing, China) were maintained in α-minimal essential medium (Gibco, Carlsbad, CA, USA) containing 10% fetal bovine serum (FBS; Gibco, Carlsbad, CA, USA) at 37 °C in 5% CO_2_ humidified air. For osteogenic differentiation, MC3T3-E1 cells were cultured in osteogenic induction medium with 10 mM β-glycerophosphate and 50 μg/mL L-ascorbic acid. For inflammation stimulation, *Escherichia coli* lipopolysaccharide (LPS; InvivoGen, San Diego, CA, USA) was added to the culture medium at a final concentration of 1 μg/mL and treated for the indicated times.

### 4.2. Cell Transfection

MC3T3-E1 cells were seeded in six-well plates and grown to 70% to 80% confluence. The cells were then transfected with 50 nM siRNA (siMETTL3, siYTHDF2, or negative control) (Invitrogen, Carlsbad, CA, USA) per well using Lipofectamine™ 3000 transfection reagent (Invitrogen, Carlsbad, CA, USA) for 12 h. A transfection rate of more than 70% of cells was used for further experiments. The siMETTL3 and siYTHDF2 sequences are listed in Table 1:

### 4.3. Real-Time Quantitative Polymerase Chain Reaction (qRT-PCR)

Total RNA was isolated from cells with RNAzol (MRC, Cincinnati, OH, USA) and reverse transcribed into cDNA using a PrimeScript^TM^ RT reagent kit (Takara, Kyoto, Japan) according to the manufacturer’s instructions. qRT-PCR was carried out with SYBR Green I Master Mix (Roche, Basel, Switzerland) using a LightCycler 480 system. The quantification of relative gene expression was performed using Gapdh as an internal control. The primer sequences are listed in Table 2.

### 4.4. Western Blotting Analysis

Cells were harvested in RIPA lysis buffer (Beyotime, Haimen, China) containing protease inhibitor cocktail (Beyotime) and phosphatase inhibitor (Beyotime). Protein concentrations were determined with a BCA protein assay kit (Beyotime). Forty micrograms of protein were separated with 8% SDS-PAGE and transferred to PVDF membranes (Millipore, Billerica, MA, USA). The membranes were blocked in 5% skim milk for 1 h at room temperature and then incubated overnight at 4 °C with the following primary antibodies: OSTERIX (1:1000; Abcam, Cambridge, UK), RUNX2 (1:1000; Abcam, Cambridge, UK), METTL3 (1:1000; Proteintech, Chicago, IL, USA), ALKBH5 (1:1000; Proteintech, Chicago, IL, USA), FTO (1:1000; Abcam, Cambridge, UK), YTHDF2 (1:1000; Proteintech, Chicago, IL, USA), p-IKKα/β, IKKα, IKKβ, p-p65, p65, p-IκBα, IκBα, p-p38, p38, p-ERK, ERK, p-JNK, JNK, p-Smad1/5/9, Smad1, and VINCULIN (1:1000; CST, Boston, MA, USA). After washing with TBST, the membranes were incubated with secondary antibodies (1:1000; CST, Danvers, MA, USA) for 1 h at room temperature. Target protein binding was developed by an enhanced chemiluminescence system (Millipore) and then scanned via an ImageQuant LAS 4000 mini system (GE Healthcare Life Sciences, Chicago, IL, USA).

### 4.5. Alkaline Phosphatase and Alizarin Red Staining

To evaluate the mineralization level, cells cultured in osteogenic medium were induced for 7 days. After washing with PBS, the cells were fixed in 4% paraformaldehyde for 20 min and then stained with alkaline phosphatase (ALP) (Yeasen, Shanghai, China) or 1% Alizarin Red S (GL Biochem, Shanghai, China) solution for 10 min. The ALP activity and matrix calcium deposition were photographed and analyzed under an inverted phase contrast microscope (Axio 40; Zeiss, Jena, Germany). The cells stained by alizarin red were incubated with 10% cetylpyridinium chloride (Sigma, St. Louis, MO, USA) for an hour. The absorbance was then measured at 562 nm.

### 4.6. mRNA Stability Measurement

Cells transfected with siRNA were cultured in osteogenic and LPS-induced medium for the indicated times and then treated with actinomycin D at a concentration of 5 μg/mL (Sigma, St. Louis, MO, USA) to inhibit global mRNA transcription. The RNA samples were collected in RNAzol at 0, 2, and 4 h. After reverse transcription, qRT-PCR was utilized to measure the mRNA degradation of the target genes.

### 4.7. Statistical Analyses

All results are presented as the mean ± standard deviation (SD) of at least triplicate experiments. The data were analyzed by Student’s *t* test or ANOVA using SPSS v20.0 software (SPSS Inc., Chicago, IL, USA). The statistically significant level was set at *p* < 0.05.

## 5. Conclusions

**Figure 6:**
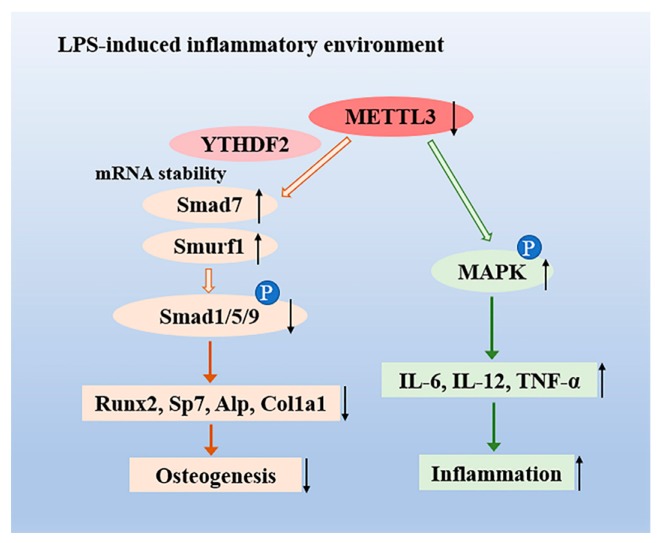
The role of the N6-adenosine methyltransferase METTL3 in an LPS-induced inflammatory model of osteoblasts. METTL3 suppression reduced the activation of Smad-dependent signaling by stabilizing the mRNA transcripts of Smad7 and Smurf1 via YTHDF2 involvement and inhibited osteoblast differentiation in an LPS-induced environment. Moreover, METTL3 depletion increased the expression of IL-6, IL-12, and TNF-α through activating MAPK signaling, thereby promoting the osteoblast inflammatory response.

In summary, we demonstrated that m^6^A methyltransferase METTL3 expression increased in osteoblast differentiation and decreased in LPS-induced inflammation. Our study illustrated that METTL3 depletion suppressed osteoblast differentiation and mineralization in both physiological and LPS-stimulated inflammatory environments. METTL3 knockdown inhibited the activation of Smad-dependent signaling and modulated the degradation of *Smad7* and *Smurf1* via YTHDF2 involvement. Moreover, METTL3 deficiency enhanced the production of proinflammatory cytokines by activating the MAPK pathway to mediate the osteoblast inflammatory response (Figure 6). These findings reveal novel insight into the epitranscriptome in osteoblast physiologic differentiation and pathological inflammation, which broadens our understanding of bone homeostasis and might provide a potential therapeutic target for bone diseases.

## Figures and Tables

**Table 1:** siRNA sequences for transcription.

siRNA	Sequences (5′-3′)
METTL3 siRNA #1	CAGCUCAGGAGUUGAUUGAGGUAAA
	UUUACCUCAAUCAACUCCUGAGCUG
METTL3 siRNA #2	UCGUUAGUCUCUGGUCUGAACUCUU
	AAGAGUUCAGACCAGAGACUAACGA
YTHDF2 siRNA	CCAUGAUUGAUGGACAGUCAGCUUU
	AAAGCUGACUGUCCAUCAAUCAUGG

**Table 2:** Primer sequences for qRT-PCR.

Gene	Forward Primer (5′-3′)	Reverse Primer (5′-3′)
*IL6*	TCTATACCACTTCACAAGTCGGA	GAATTGCCATTGCACAACTCTTT
*IL12*	GTCCTCAGAAGCTAACCATCTCC	CCAGAGCCTATGACTCCATGTC
*TNF-a*	CCACCACGCTCTTCTGTCTA	GGTCTGGGCCATAGAACTGA
*Runx2*	TTCAACGATCTGAGATTTGTGGG	GGATGAGGAATGCGCCCTA
*Sp7*	ATGGCGTCCTCTCTGCTTG	TGAAAGGTCAGCGTATGGCTT
*Alpl*	GGCTGGAGATGGACAAATTCC	CCGAGTGGTAGTCACAATGCC
*Col1a1*	CCCTGCCTGCTTCGTGTA	TTGAGTTTGGGTTGTTCGTC
*Mettl3*	CTTTCTACCCCATCTTGAGTG	CCAACCTTCCGTAGTGATAGTC
*Fto*	GACTCGTCCTCACTTTCATCC	AAGAGCAGAGCAGCCTACAAC
*Alkbh5*	GTGGGACCTTTTGGGTTTCAG	GCATACGGCCTCAGGACATTA
*Ythdf2*	ATAGGAAAAGCCAATGGAGGG	CCAAAAGGTCAAGGAAACAAAG
*Smad7*	GGGCTTTCAGATTCCCAACTT	AGGGCTCTTGGACACAGTAGA
*Smurf1*	ACACTGGCTACCAGCGTTTG	TCTGTCTCGGGTCTGTAAACTG
*Gapdh*	GGTCATCCCAGAGCTGAACG	TGCTGTTGAAGTCGCAGGA
